# Monitoring System for Farming Operations with Wearable Devices Utilized Sensor Networks

**DOI:** 10.3390/s90806171

**Published:** 2009-08-04

**Authors:** Tokihiro Fukatsu, Teruaki Nanseki

**Affiliations:** 1 National Agricultural Research Center, National Agriculture and Food Research Organization/3-1-1 Kannondai, Tsukuba, Ibaraki 305-8666, Japan; 2 Faculty of Agriculture, Kyushu University/6-10-1 Hakozaki, Higashi, Fukuoka 812-8581, Japan; E-Mail: nanseki@agr.kyushu-u.ac.jp

**Keywords:** automatic recognition, farming operation, wearable device, RFID, Field Server

## Abstract

In order to automatically monitor farmers’ activities, we propose a farm operation monitoring system using “Field Servers” and a wearable device equipped with an RFID reader and motion sensors. Our proposed system helps in recognizing farming operations by analyzing the data from the sensors and detected RFID tags that are attached to various objects such as farming materials, facilities, and machinery. This method can be applied to various situations without changing the conventional system. Moreover, this system provides useful information in real-time and controls specific machines in a coordinated manner on the basis of recognized operation.

## 1. Introduction

It is important to monitor crop growth, field environment, and farming operations in order to increase the agricultural productivity and to promote efficient management. However, it is difficult to realize these monitoring operations automatically in the agricultural field because it requires the deployment of specialized equipment and the improvement of facilities that require considerable effort, space, and cost. A key technology to assist in field monitoring is a sensor network [1,2], with each sensor equipped with a radio link. We have developed a Field Server [3] as a sensor network for agricultural use. A Field Server enables crop and environmental monitoring by using various sensors and cameras, but it is insufficient for obtaining detailed information about farming operations. The data for farming operations, especially manual tasks, can be recorded using several approaches such as writing them manually, inputting them by using an assistant software, and monitoring them with IT tools. In order to apply data-input systems on an agricultural field, some systems using Internet cell-phones and PDAs have been developed [4–8]. These methods cannot be easily applied for practical purposes because operating these computers are a troublesome task for farmers, especially the elderly, and the implementation of these methods requires farmers to interrupt their field operations to input data. Other systems equipped with GPS or voice entry have also been developed to solve this problem [9–11]. These methods handle only general items such as pesticide spraying and it is difficult to allow flexible use; however, monitoring farmers’ activities in detail including what they observe, which pesticide they choose, in what area they operate, and how much they spray, is desirable in order to realize a more useful and effective support system.

**Figure 1.**
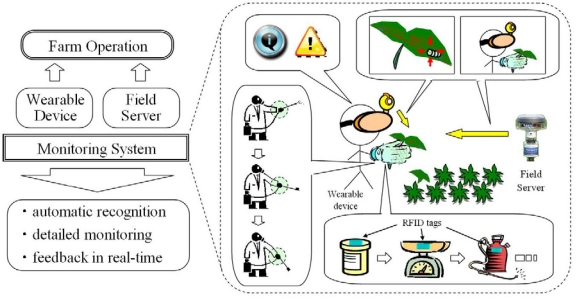
Concept of farm operation monitoring system.

In this study, we propose an innovative farm operation monitoring system to solve these problems by using the Field Server system and a wearable device that we developed; this device is equipped with a radio frequency identification (RFID) reader and motion sensors (Figure 1). In this system, we recognize a farmer’s operation automatically under various situations by analyzing the data from the sensors and the RFID tags, which are attached to all relevant objects such as farming materials, facilities, and machinery. Our proposed system is developed on the basis of the Field Server system that provides not only various monitoring operations and peripheral controlling but also network infrastructure, effective management system and extensible architecture to develop service applications. In this paper, we explain the concept and feature of the system and then evaluate its effectiveness and potential through several experiments using a prototype system.

## 2. Farm Operation Monitoring System

### 2.1. Field Server System

**Figure 2.**
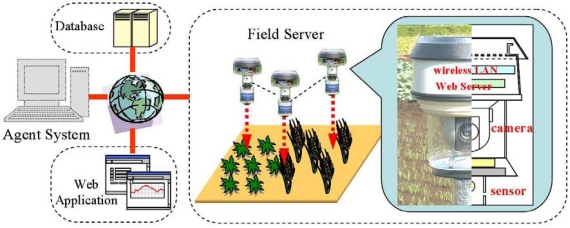
System architecture of Field Server.

A Field Server, a web-based sensor node of an advanced sensor network system, has a wireless LAN, an Internet camera, and a monitoring unit with a Web server (Figure 2). By controlling and measuring various sensors including the camera, we can monitor not only the field environment but also crop growth, insect infestation, and simple field operations [12]. A wireless LAN provides high-speed transmission and long-distance communication at low cost. Therefore, a Field Server is effective in collecting high-resolution image data. It also provides network infrastructure and a hotspot area in the agricultural field in order to overcome the digital divide.

Each module of the Field Server can be accessed through a Web page using a Web browser such as Internet Explorer. It performs remote operations and monitoring with a management program called Agent System [13]. An Agent System manages all types of Web-based modules via the Internet, and so the Field Server can be developed with a simple firmware on the inside. An Agent System can choose its operations flexibly and autonomously according to users’ requests and changing situations with a rule-based function. It can also analyze the monitored data in real-time in collaboration with useful Web applications such as image analysis and signal processing [14]. This architecture provides versatile and easily expandable functions without changing or rebooting the main program and makes it possible to distribute calculation tasks.

By developing the farm operation monitoring system based on the Field Server system, we can exploit some of its advantages. Network infrastructure in the agricultural field is available to the monitoring system, and so a simple and compact wearable device fit for farmers can be developed by separating some functions via the network. This proposed system can be easily managed and can be applied to the complicated recognition method with an Agent System by using a wearable device consisting of Web-based modules. Some support applications in response to farming operations are also provided easily by using the distributed Web processing function and peripheral control units embedded in Field Servers.

### 2.2. Concept

The concept of our farm operation monitoring system is to provide versatile, scalable, and user-friendly monitoring system which recognizes users’ behavior in detail under various situations. To develop a useful monitoring system introduced the concept, it is important to consider the following requirements:

- The system should not encumber the farmer’s activities during farming operations.
- It should be easy to use for non-experts and should not involve complicated processes.
- It should be available under many conditions without changing the facilities and equipment.
- It should monitor detailed farming operation and various conditions such as plant growth.
- It should make effective use of monitoring information in real-time.

In order to achieve these requirements, we propose a recognition method for farming operation with a wearable device equipped with an RFID reader and motion sensors. The basic function of RFID is to provide object identifications and the RFID system is generally used for individual recognition in some areas of logistics, security control, traceability system, and livestock industry [15–18]. In the livestock industry, RFID tags are attached to or in animal bodies and some applications such as health control, fattening management, milking management, and tracking behavior are implemented by checking the measurement data against detected RFID tags [19–21]. In our system, however, we adapted it for use in the recognition of farming operations. The prototype method of this system was discussed in our previous research [22] and this proposed system was constructed to be expanded to various operations in farming locations based on the prototype method.

RFID tags are attached to relevant objects such as farming materials, implements, machinery, facilities, plants, and fields. A farmer performs farming operation with a wearable device and it detects a sequence of these RFID tags throughout the farmer’s activities. In this proposed system, the farmer’s operations are deduced by analyzing a combination of patterns of detected RFID tags and the change in motion sensing data. In the conventional applications, RFID tags are attached to objects which themselves are important targets to be observed. In our system, however, a farmer puts on not an RFID tag but an RFID reader in order to apply this system to various operations easily. Also in this system, not only a single piece of detected tags but also a series of detected ones are utilized to derive the desired information, unlike the conventional applications.

This method is flexible and available under many conditions without changing the facilities or equipment. All that is required is to attach RFID tags to existing objects and to perform farming operation with a wearable device. For example, we can monitor how a farmer operates agricultural implements by analyzing the data of motion sensors and RFID tags that are attached to parts of implements such as handles, levers, buttons, and keys. By attaching RFID tags to plant trays, partitions, and fields, we can also monitor the time and locations of operations in a greenhouse where GPS sometimes does not function well. In some situations such as the distinction between holding an implement and using it, it is difficult to detect the farming operation only by the data of RFID tags. The sensing data is used for an interpolated data of RFID tags and for recognizing detailed operations. This method recognizes farming operations more accurately and specifically by using both motion sensors and RFID tags.

In the proposed system, detected data from the wearable device is analyzed at a remote site instead of by an internal computer. By separating this function, the wearable device becomes a simple, small, and lightweight unit with high performance. By using the detected pattern and sensing data, farming operations are recognized with estimation algorithms such as pattern matching, Bayesian estimation, principal component analysis, and support vector machines which classify the data in groups of farming operation with supervised learning. This recognition function is easily and effectively modified because of the distributed processing architecture. This distributed processing architecture also enables interactive support applications. By using Field Servers and peripheral devices, these applications control suitable machines in a coordinated manner, monitor various conditions in detail, and provide useful information to a farmer in response to recognized farming operations.

With the feature of this system, we can also provide potential benefits to help some farming system such as schedule management, decision support, and traceability. This system enables to obtain the information about the progress and speed of farming operation which is important items for an efficient schedule and labor management [23]. One of the problems with a decision support system is a shortage of case data and this system offers a possible solution to collect enormous amount of data including not only farming operation but also crop growth and field environment [24]. This system also enables to automatically record the information about irrigation, fertilization, and other cultural treatments. In the upside of traceability, this information is recently required and this system is expected as one of the solutions to implementing traceability, along with auto-control machines [25]. Especially, this system is effective in situations that a farmer manually performs the cultivation management.

### 2.3. System Design

**Figure 3.**
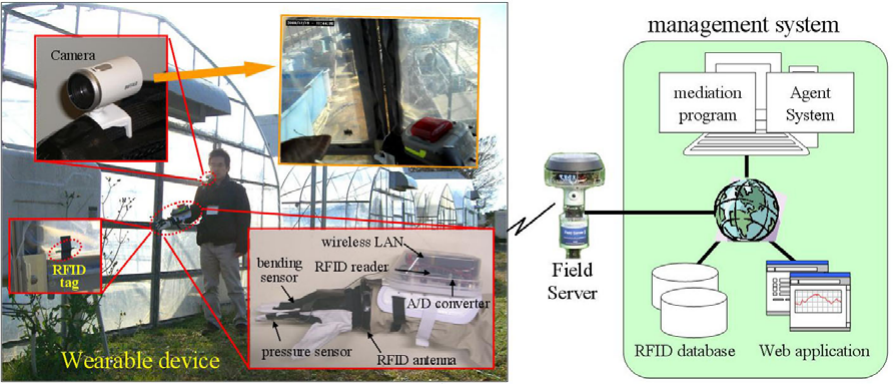
Architecture of farm operation monitoring system.

Figure 3 shows the architecture of the farm operation monitoring system. It includes components such as RFID tags, a wearable device, Field Servers and a management system. In the field site, Field Servers are deployed and RFID tags are attached to object items which have possibilities to be contact with a farmer in the process of operations. The information of the object items and attached RFID tags is preliminarily registered into the RFID database in the management system. There are some types of RFID tags with different frequencies (2.45 GHz, 13.56 MHz, and 134.2 kHz) that differ in terms of communication distance, tag shape, antenna size and regulation [26]. In this study, the 134.2-kHz-type of RFID is used because of the emphasis on the communication distance.

The wearable device is equipped with a wireless LAN for connecting a management system, an RFID reader for detecting relevant objects, and an A/D converter with sensors for monitoring a farmer’s motion. In order to make the wearable device useful, some features such as comfortable fit, unimpeded body, suitable sensors, and sufficient sensitivity are required. In this study, we developed a prototype wearable device consisting of a micro reader (RI-STU-MRD1, Texas Instruments) with a modified antenna as an RFID reader, an electric circuit with a microcomputer (PIC16F877, Microchip Technology) as an A/D converter, and a device server (WiPort, Lantronix) wired to the micro reader and the electric circuit. This device server also incorporates a wireless LAN. The A/D converter has four input channels and the pressure sensor for the farmer’s hand is connected to it. This wearable device works for up to two hours when a set of four AA batteries is used. The battery life can be extended by using energy-saving units and modifying the always-on management. In some experiments, a network camera unit for collecting user-viewed image data and a wearable computer display unit for providing useful information are added to the system.

**Figure 4.**
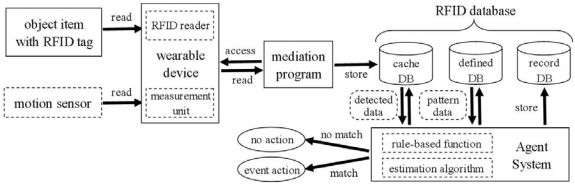
Block diagram of the management system.

Figure 4 shows the block diagram of the management system which analyzes the detected data, recognizes farming operations, and executes appropriate actions in response to the results at a remote site. The RFID reader and a measurement unit are accessed via Field Servers by a mediation program in the management system. The mediation program collects sensor and tag data from the wearable device and stores them in the database (using Microsoft Access). An Agent System does not access the wearable device directly but rather accesses this database as a cache because the database is able to efficiently treat a vast amount of data stored at high frequency (200 ms). After accessing the database, the Agent System recognizes farming operations with an estimation algorithm. In this study, we simply choose pattern matching with defined pattern table as an estimation algorithm.

An Agent System with rule-based functions and distributed Web processing functions can also provide support applications in response to farming operations (event action). Some existing Web applications such as a navigation system for appropriate pesticide use [27] are available for this system in order to provide useful information to farmers. Some field devices connected to Field Servers can be appropriately controlled to assist farming operations using the Agent System. In this case, it is necessary to consider avoiding controlling the interference between this event action and scheduled operation. In order to solve this problem, we introduced a multi Agent System [28] in which one agent exclusively performs the scheduled operation and the other agent performs event action in a coordinated manner.

## 3. Experiments

### 3.1. Performance of RFID Reader

In order to perform the automatic recognition of farming operations with the wearable RFID reader, it is important to detect RFID tags with a high degree of accuracy without any conscious action. In our previous experiments, two kinds of RFID systems (2.45 GHz and 13.56 MHz) were required to bring a reader into contact with the tags consciously [22]. Given the various operation contents and tag-attached situations, a high-performance antenna that enables us to read RFID tags without contact is required in this system. We have developed three types of antennas and used them to evaluate the system’s performance in terms of communication distance.

**Figure 5.**
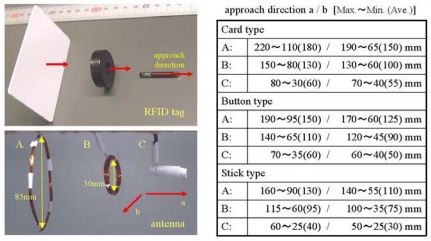
Experimental materials and results of RFID accessible distance.

Figure 5 shows the experimental results and materials such as typical RFID tags (card, button, and stick type) and antennas [bracelet- (A), ring- (B), and fingertip-type (C)] considered to have an easily wearable shape and adequate inductance of the antenna coil (47 uH for 134.2 kHz). In this experiment, we measured the accessible distance between the tags and the antennas from the two directions [at vertical (a)/horizontal (b) to the normal line of magnetic force] ten times each. These tags were attached to objects and an operator performed the experiments with each antenna. The bracelet-type antenna has sufficient distance (average: 110 – 180 mm) for each tag under various situations. The communication distance of the fingertip-type antenna is about one-third that of the bracelet-type, but it is capable of detecting only fingered objects selectively. Tag type has an insignificant effect on detecting performance under this condition. This result shows that all combinations of tags and antennas are better than the other types of RFID (2.4 GHz, 13.56 MHz) whose accessible distance is a few centimeters according to manufacturer’s specifications.

### 3.2. Recognition of Farming Operation

To evaluate the basic performance of this system in a field environment, we performed three fundamental experiments in farming operation locations. One experiment was the recognition of transplanting operation in which a user took each potted seeding, checked the seeding condition and transplanted it to a larger pot if it grew well. In this experiment, RFID tags were attached to every pot and a user performed the operation with a wearable device. We arranged twelve potted seeding including two bad ones and tested the detailed information about this operation was able to be obtained by using our proposed system.

**Figure 6.**
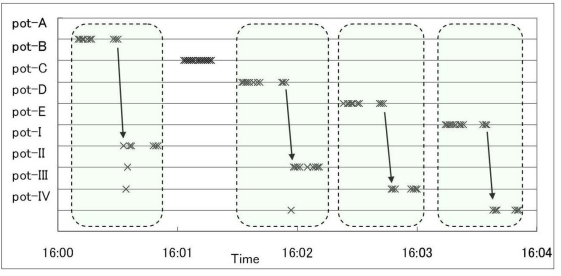
Recognition result of transplanting operation.

Figure 6 illustrates some results from the experiment. The cross mark shows the detected RFID tags corresponding to each pot. The pot-A to pot-E (smaller pot) were potted seeding and the pot-I to pot-IV (larger pot) were empty pots for transplanting. Every target pot which a user touched during the operation was identified without any problem, and the transplanting operation was recognized with the defined pattern of detecting a larger pot after a small pot for over three seconds. When a user took a larger pot, an RFID tag of another larger pot was mistakenly detected several times because these larger pots were piled up. By using the mode value of detected RFID tags during a few seconds, this system accurately recognized the operation and the correspondence relation between the smaller pot and the larger pot transplanted the target seeding was able to be obtained (e.g., the seeding in a pot-A, -C, -D and -E were transplanted to the pot-I, -II, -III and –IV). In this experiment, a user didn’t transplant the pot-B because of the bad seeding, so the pot-B didn’t have the corresponding larger pot. By using this information, we were able to obtain the classification result of seeding. In this system, not only detected RFID tag identification number but also the detected time was stored in the database. By using the detected time, this system was able to provide the detailed information such as the speed of the operation calculated by subtracting the first time of detected the smaller pot from the last time of detected the larger pot.

The next experiment was the recognition of entering and leaving of greenhouses by using the detected patterns of RFID tags. In this experiment, RFID tags were attached to both sides of the door (tag-A: outside; tag-B: inside). A user equipped with a wearable device worked inside and outside greenhouses and this system detected the entering and leaving by performing pattern matching of the sequence pattern of the detected tags.

**Figure 7.**
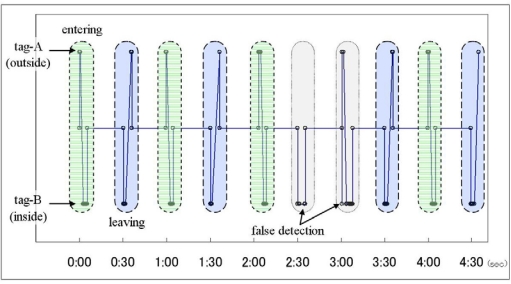
Recognition result of entering and leaving.

Figure 7 illustrates some results from the experiment, which tested the recognition of both entering and leaving two kinds of greenhouses eight times each. In this estimation algorithm, the entering operation is defined as a pattern of detecting tag-B a few seconds after tag-A and the leaving operation is defined as an opposite pattern. By using our proposed system, we can distinctly detect the entering and leaving actions; the percentage of accurate recognition in total was 87.5% for entering and 81.3% for leaving. The main reason for misrecognition was not the detection error due to inadequate antenna sensitivity but false detection due to excessive antenna range, in which the antenna mistakenly detects a far-side tag through the door.

The third experiment is the recognition of the cutoff operation with a pair of scissors. In this experiment, RFID tags were attached to each plant tray and to the handle of scissors. In order to recognize the time for cutting a branch, this system made judgments with the data of RFID tags and the pressure sensor for the forefinger. A user equipped with a wearable device and a network camera unit on the shoulder cut the designated branches and the system collected a user-viewed image data during each cutoff operation after recognizing the operation.

**Figure 8.**
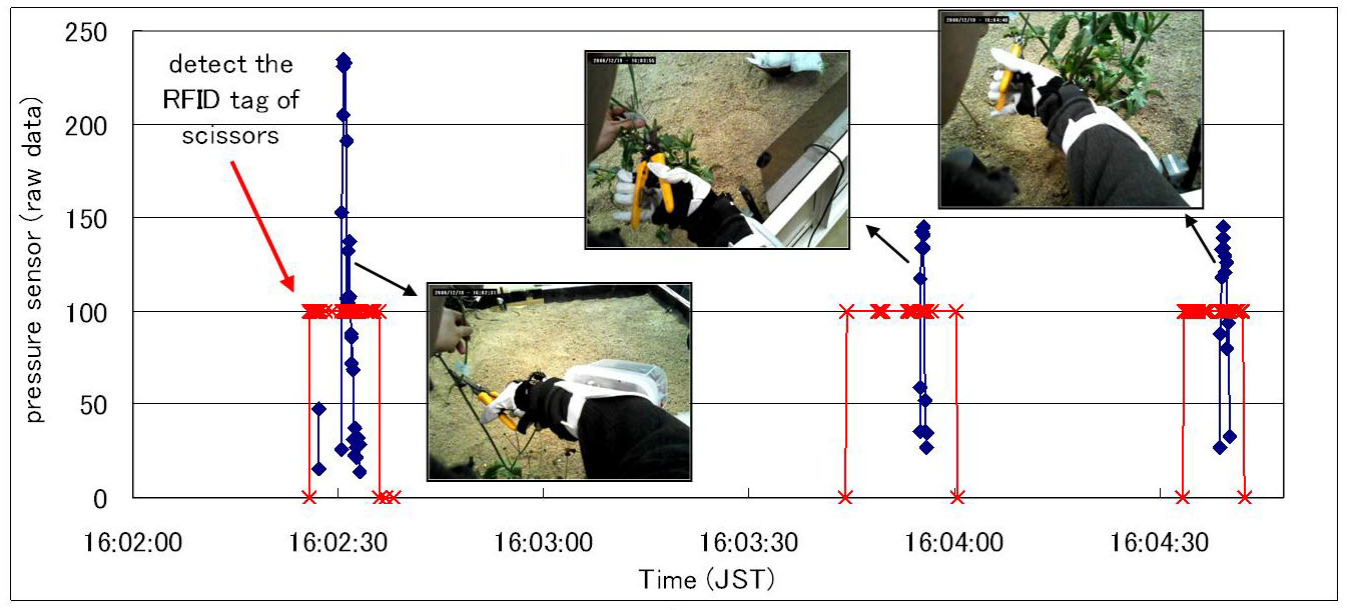
Recognition result of cutoff operation.

Figure 8 illustrates some results from the experiment, which tested the cutoff operation five times each in two kinds of plants. While the value of the pressure sensor exceeded the threshold (preliminarily calibrated) and the RFID tag of scissors was detected after the RFID tag of the plant tray, the image data was collected. In this system, the state of holding or using the scissors was fully recognized and the percentage of accurate recognition was 80% in total. The main reason for the misrecognition was that the value of the sensor did not exceed the threshold because the position of the sensor attached to a glove was not accurate for a user.

### 3.3. Support Application According to Farming Operation

This system enables us not only to recognize farming operations but also to provide support applications in response to the recognized operation. In this experiment, we developed support applications for the farming operation of pesticide preparation, in which it is important for the farmer to provide useful information regarding proper usage and to record the operation procedure for control of pesticide and traceability. RFID tags were attached to a warehouse door, some points of a rack in a warehouse and stored pesticide bottles, and a Field Server equipped with a controllable camera was deployed near the warehouse. When the system recognized that a certain bottle was being taken, it accessed the camera of Field Server to record the target process with a zoom function, and it also sent detailed information and judgment for appropriate use regarding the target pesticide to a wearable computer display.

**Figure 9.**
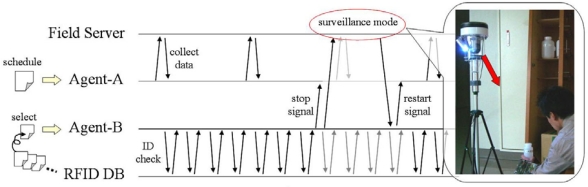
Operation status flow of Agent System.

Figure 9 shows the operation status flow of the management system in this experiment. One agent (Agent-A) monitored the Field Server on the basis of its scheduled operation and the other agent (Agent-B) periodically checked the RFID database. When a defined operation was recognized, Agent-B sent a stop signal to Agent-A in order to avoid access collision, and Agent-B preferentially directed the camera of the Field Server to the position of the detected rack-attached RFID tags. When a user with a wearable device tried to bring out the bottles randomly in this experiment, the system was able to record the target operation procedure as the image data without problems.

**Figure 10.**
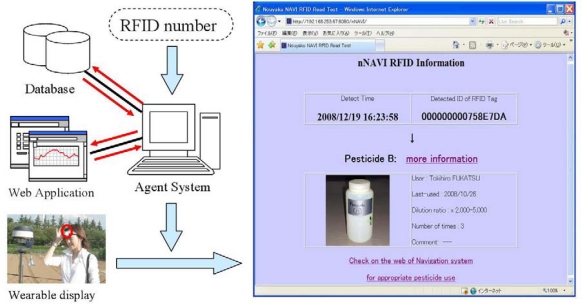
Support application of providing useful information.

In this experiment, we also tested whether the system was able to provide appropriate information. After the recognition of the operation, Agent-B searched for the pesticide name and detailed information in a prepared list. This information was converted into an HTML file including a link to the judgment for the appropriate pesticide use provided by the Web application (Figure 10). In this experiment involving five kinds of pesticide bottles, the user was able to obtain correct information in the HTML format on a wearable computer display connected to the Internet via the Field Server.

## 4. Discussion and Conclusions

We have proposed a farm operation monitoring system consisting of Field Servers and a wearable device and evaluated the potential and effectiveness of this system. These experiments show that the system is able to recognize farming operations well and to provide support applications in response to the operation. Through the experiments, we determined the future directions of research on wearable device, recognition method, and practical system.

In the recognition experiment, there were some failed detections of RFID tags because of excessive antenna sensitivity. We propose solutions such as adequate range design with directional antennas, a multi-antenna switching method with a phased array, and a tag allocation technique that is based on objects and operations [29,30]. In this study, we attempted to recognize complex and detailed operations using an RFID reader and pressure sensors as a motion sensor. By using many kinds of motion sensors such as finger bending sensors, acceleration sensors, and capacitive sensors, this system will recognize farming operation with a high degree of accuracy under various situations.

In order to recognize farming operations more accurately, it is required not only is correct detection of RFID tags on the objects required, but also the algorithm for the estimation of farming operations from detected data must be improved. We can choose many kinds of estimation algorithms, but the algorithm should be customized and adjusted on the basis of the performance of the wearable device, tag allocation, operation contents, and user requirements.

This system enables one to record a farmer’s operation easily and automatically. It is effective to realize precision management of cultivation in some situations with manual tasks such as protected horticulture, fruit cultivation, and small-scale farming for which it is difficult to perform mechanized farming. In food traceability, not only supply chain but also farmers are required to record the processing of products [31]. The record of cultivation management, especially pesticide use, has become increasingly important, but the task requires much effort. For a farmer enforced through legal requirements, this system is helpful to establish traceability and to provide detailed information such as image data. This system also enables one to monitor the plant which is involved certain operations. By using the information of operation content and plant growth, we can develop a specific database of farming systems which helps to understand the effect of operation, field condition, and farmer’s criteria.

In this study, we only developed support applications to provide information and to record operations; however, this system enables us to apply interactive applications such as sharing databases regarding operation techniques, controlling assist tools, and having navigation- and attention-system related to operations. If we are able to obtain information not only on farming operations but also on the farmer’s behavior, e.g., what he pays attention to and how he interacts with this system, the database stored by our system will become an important tool for understanding farmers’ practices. The navigation- and attention-system, which is one of the decision support system, provides the useful information such as the tutorial of next operation, concerned data for the judgment, and the warning of misuse to a farmer in real-time. It will enhance farmer’s sensitivity, judgment, and activity by using the farm operation monitoring system. From the viewpoint of the Field Server system, we can treat the wearable device as a special Field Server that selectively measures important objects with mobile fields. In conventional monitoring with an automatic system, we were not able to obtain these measurement data. By improving the system, we can develop the new concept of a user-based monitoring system in a sensor network and this system can be applied for not only in agriculture but also in the other fields.
